# Additive Neuroprotective Effect of Borneol with Mesenchymal Stem Cells on Ischemic Stroke in Mice

**DOI:** 10.3389/fphys.2017.01133

**Published:** 2018-01-17

**Authors:** Xiao-Guang Zhang, Chang Shan, Jia-Zhen Zhu, Xiao-Yi Bao, Qiang Tong, Xi-Fan Wu, Xiao-Chen Tang, Ting Xue, Jie Liu, Guo-Qing Zheng, Yan Wang

**Affiliations:** ^1^Department of Internal Medicine, The Second Affiliated Hospital and Yuying Children's Hospital of Wenzhou Medical University, Wenzhou, China; ^2^Translational Center for Stem Cell Research, Tongji Hospital, Stem Cell Research Center, Tongji University School of Medicine, Shanghai, China; ^3^Department of Endocrine and Metabolic Diseases, Shanghai Clinical Center for Endocrine and Metabolic Diseases, Rui-jin Hospital, Shanghai Jiao-tong University School of Medicine, Shanghai Institute of Endocrine and Metabolic Diseases, Shanghai, China

**Keywords:** mesenchymal stem cells, borneol, stroke, neurogenesis, neuroprotection

## Abstract

Intravenous stem cell transplantation initiates neuroprotection related to the secretion of trophic factor. Borneol, a potential herbal neuroprotective agent, is a penetration enhancer. Here, we aimed to investigate whether they have additive neuroprotective effect on cerebral ischemia. Borneol was given to mice by gavage 3 days before middle cerebral artery occlusion (MCAO) induction until the day when the mice were sacrificed. Mesenchymal stem cells (MSCs) were intravenously injected at 24 h after MCAO induction. Neurological deficits, infarct volume, cell death, and neurogenesis were evaluated. Combined use of MSCs and borneol could more effectively reduce infarction volume and cell apoptosis, enhance neurogenesis, and improve the functional recovery than that of MSCs alone. The findings showed that combined use of borneol and stem cells provided additive neuroprotective effect on cerebral ischemia. However, the supposed effect of borneol on the improved MSC penetration still needs further direct evidence.

## Introduction

Ischemic stroke is one of leading causes of adult death and physical disability worldwide (Kleinschnitz et al., 2010). Its morbidity and mortality keep increasing during last decades especially in developing countries, which brings enormous social and economic burden to patients and their caregivers (Hao et al., 2014). Despite considerable progress, including the establishment of a specialized stroke care unit, thrombolysis with tissue plasminogen activator within 4.5 h after the stroke attack, endovascular treatment of acute ischemic stroke and prevention therapies for secondary stroke, few neuroprotective therapies exist which can effectively reduce brain damage and improve neurological recovery.

Stem cell-based therapy has emerged as a novel and promising candidate approach by the limited eligibility for thrombolysis and failure of the neuroprotective paradigm (Jäkälä and Jolkkonen, 2012). Although advantageous effects of intracerebral delivery of stem cell in stroke could be obtained from high intracerebral numbers of grafted cells, the process of invasive surgery was not suitable for clinics (Doeppner et al., 2012). Intravenous stem cell transplantation was reported safe and might improve recovery for stroke patients (Bang et al., 2016). Unfortunately, studies in rodents have clearly shown that following intravenous administration, stem cells accumulated mainly in the lungs, liver, spleen, and bones, whereas the concentration reached in the brain is negligible (Bansal et al., 2015; Ohshima et al., 2015). Thus, intravenous mesenchymal stem cells (MSCs) initiates neuroprotection, which might secrete various bioactive substances into the injured lesions such as trophic factors and extracellular vesicles, leading to enhanced neurogenesis, angiogenesis and synaptogenesis (Chen et al., 2002; Liu Z. et al., 2011). Although these nutritional ingredients could penetrate the damaged blood-brain barrier (BBB) to some extent in condition of ischemic stroke, the concentration accumulated in the brain might not be high enough to produce sufficient curative effect.

Borneol, a terpene and bicyclic organic compound found in several species of Artemisia and Dipterocarpaceae, has shown many bioactivities such as anti-inflammatory, improving energy metabolism and neuroprotection against cerebral ischemia/reperfusion injury (Gutiérrez-Fernández et al., 2011; Ehrnhofer-Ressler et al., 2013). Furthermore, its strong fat-soluble active ingredients made it a quite good penetration enhancer. Several lines of evidence have revealed that borneol could increase the concentration of other agents in brain tissues such as gastrodin and edaravone (Cai et al., 2008; Wu et al., 2014). The penetration enhancing effects of borneol can be acted via the reaction of the lipophilic fraction with the lipid parts of cell membranes, and thus influence the bioactivity of enzymes, carriers, ion channels, and receptors (Gabbanini et al., 2009). Borneol also had the ability to increase the number and volume of pinocytosis vesicles in BBB cells, and thus accelerate the transportation of substance by way of cell pinocytosis in the brain (Chen et al., 2010). Currently, borneol has attained increasing attention as a promising approach in nervous system diseases as disparate as stroke (Liu R. et al., 2011), Alzheimer disease (Han et al., 2011), and Parkinson's disease (Tian et al., 2007).

Given these benefits between borneol and stem cells, a hypothesis was raised that combined use of borneol and stem cells might bring more benefits for ischemic lesions, obtaining an additive neuroprotective effect. Thus, in the present study we established ischemic mouse model through middle cerebral artery occlusion (MCAO) and then evaluated the effect of borneol, C57BL/6 mouse fetus-derived MSCs and borneol plus MSCs on neurological deficits, infarct volume, cell death and neurogenesis, respectively.

## Materials and methods

### Animals

Eighty adult male C57BL/6 mice, 11–13 weeks of age, body weight 25–30 g, were obtained from Shanghai laboratory animal research center and housed in the experimental animal center of Shanghai Tongji hospital. All the mice were housed under 12 h light/dark cycles, temperature 22 ± 1°C and provided with available food and water *ad libitum*. All procedures were performed following the national institutes of health for the care and use of laboratory animals (NIH publication No. 8023) and were approved by the animal ethics committee of Shanghai Tongji hospital. All efforts were made to minimize the suffering of animals used and the number of animals needed for this study.

### MSCs isolation and identification

MSCs were isolated and harvested as follows. Briefly, fetal mice were obtained by aseptic cesarean delivery from 13.5 d pregnant mice and flushed with phosphate-buffered saline (PBS). The body of fetal mice was clearly isolated and cut into pieces <1 mm^3^, followed by trypsinization at 37°C for 5 min, with DMEM used to neutralize trypsin activity. The suspension was centrifuged, resuspended with Dulbecco modified Eagle medium (DMEM, Gibco) supplemented with 10% fetal bovine serum (FBS, Gibco) and cultured in 10 cm dish at 37°C with 5% CO_2_ in an incubator. Nonadherent cells were removed every 48 h and primary cells were subcultured 1:3 when 80%-90% confluence. On the third pass (P3) they were trypsinized and counted before administration to the experimental mice.

Identification of MSCs was performed by flow cytometry to examine the surface markers CD11b-PE (1:200, Biolegend), CD29-FITC (1:200, Biolegend), CD34-FITC (1:200, Biolegend), CD44-PE (1:200, Biolegend), CD45-APC (1:200, Biolegend), and SCA1-APC (1:200, Biolegend). Cells (1 × 10^6^) were incubated in a 200 μl buffer (PBS with 1 μl of CD11b-PE, CD29-FITC, CD34-FITC, CD44-PE, CD45-APC, SCA1-APC, and isotope control antibodies, respectively) for 30 min at 4°C in the dark followed by three washes with PBS. Next, the cells were resuspended in 200 μl PBS and analyzed in an FACS instrument (BD AccuriC6).

### Osteogenic and adipogenic induction of MSCs

On the third pass (P3) with 60–80% confluence the cells were trypsinized and plated into 12-well plates at a density of 1 × 10^4^/mL. The cells were cultured in DMEM supplemented with 10% FBS, 100 U/mL penicillin, 1 mg/mL streptomycin in combination with either osteogenic induction medium, including dexamethasone (0.1 μmol/L), β-sodium glycerophosphate (10 mmol/L) and ascorbic acid phosphate (50 μmol/L), or adipogenic induction medium, including dexamethasone (1 μmol/L), insulin (5 or 10 mg/L), IBMX (0.5 mmol/L) and indomethacin (200 μmol/L). The medium was replaced every 3 days for 2 weeks. Calcium deposits and fat droplet formation were observed by Alizarin Red S staining and oil red O staining, respectively.

### Focal cerebral ischemia-reperfusion model and agents administration

Transient MCAO model was employed as focal cerebral ischemia/reperfusion model following a previously described method (Haddad et al., 2013). The filament was removed after 1 h occlusion to induce reperfusion. Sham group received the same procedure without the induction of the filament. Mice receiving surgery showing a Longa score from 1 to 3 points at 6 h post-stroke were included in this study (Longa et al., 1989). Those animals that showed lack of neurologic deficit (*n* = 4), brain hemorrhage (*n* = 3), or death after ischemia (*n* = 5) were excluded from the study. Animals were randomly divided into the following 5 groups (*n* = 16 per group): Sham, MCAO plus saline, MCAO plus borneol, MCAO plus MSCs and MCAO with borneol plus MSCs.

At 24 h after MCAO induction, animals were anesthetized with 1% sodium pentobartital and then subjected to intravenous injections of 5 × 10^5^ MSCs in 0.2 ml sterile PBS solution through caudal vein. MCAO plus saline group received the same procedure except for the injection of the same amount of saline. Borneol was dissolved in 5% Tween 80 and given to mice by gavage at 200 mg/kg 3 days before MCAO induction until the day when the mice were sacrificed.

### Neurological deficits evaluation

Neurological deficits were evaluated by global score of neurological score (Wahl et al., 1992) as well as grip and string tests (Haddad et al., 2013), which were performed in a dedicated room at 20–22°C on 1st, 3rd, 7th, and 14th day after MSCs transplantation. The scores of the three tests were added as the global score, the maximum of which was 21. The lower global score indicated the more severe neurological deficits.

### Cerebral infarct volume measurement

At 7 days after MSCs transplantation mice were sacrificed. The brain was removed and then coronally sliced into 1 mm slices. These slices were stained with 2% 2,3,5-triphenylterazolium (TTC) in normal saline for 30 min at 37°C. Normal brain tissue was stained red while the unstained area was considered to be the infarct area. The size of infarct areas were measured by Image J software (National Institutes of Health, Bethesda, USA) and presented as the percentage of the total brain volume.

### Cell death

Apoptotic cell death was detected in 6 μm frozen coronal sections by terminal deoxynucleotidyl transferase dUTP nick end labeling (TUNEL) staining (Roche Diagnostics, Mannheim, Germany) following the manufacturer's protocol. Sections were counterstained with DAPI. The number of TUNEL-positive cells in infarct zone was counted Image J software (National Institutes of Health, Bethesda, USA).

### Immunofluorescence staining

Two weeks after MSCs transplantation, coronal sections (6 μm) were processed for immunofluorescence staining. Sections were permeabilized with Triton X-100 and blocked with goat serum, then incubated with primary antibodies overnight at 4°C, either mouse anti-NeuN: (1:500; Millipore) or rabbit anti-GFAP: (1:1,000; Dako). Second antibodies including goat anti-rabbit or goat anti-mouse secondary antibody-conjugated to Alexa594 (1:500; Gibco) were used at 37°C for 1 h. Immunofluorescent signaling was observed with an Olympus fluorescence microscope (Olympus, Tokyo, Japan). The number of NeuN-positive cells were counted blindly using Image J software (National Institutes of Health, Bethesda, USA) and the integral optical density (IOD) of GFAP was obtained using Image-ProPlus 4.1 software (Media Cybernetics, Rockville, MD, USA).

### Statistical analysis

Data were expressed as mean ± standard deviation (*SD*). Normality was determined using the Kolmogorov–Smirnov test. If normality was given and there were no significant differences in variance between groups (*F*-test), multiple groups were compared using one-way analysis of variance (ANOVA) and followed by LSD post hoc comparisons when appropriate. Otherwise, comparisons for non-normally distributed data were performed using non-parametric Kruskal–Wallis test followed by Mann–Whitney *U*-test. All statistical analyses were performed using SPSS 20.0 software (IBM, Armonk, NY) and GraphPad Prism 6.05 (GraphPad, La Jolla, CA). Values of *P* < 0.05 were considered statistically significant.

## Results

### Characterization of mouse fetus-derived MSCs

**Figure 1:**
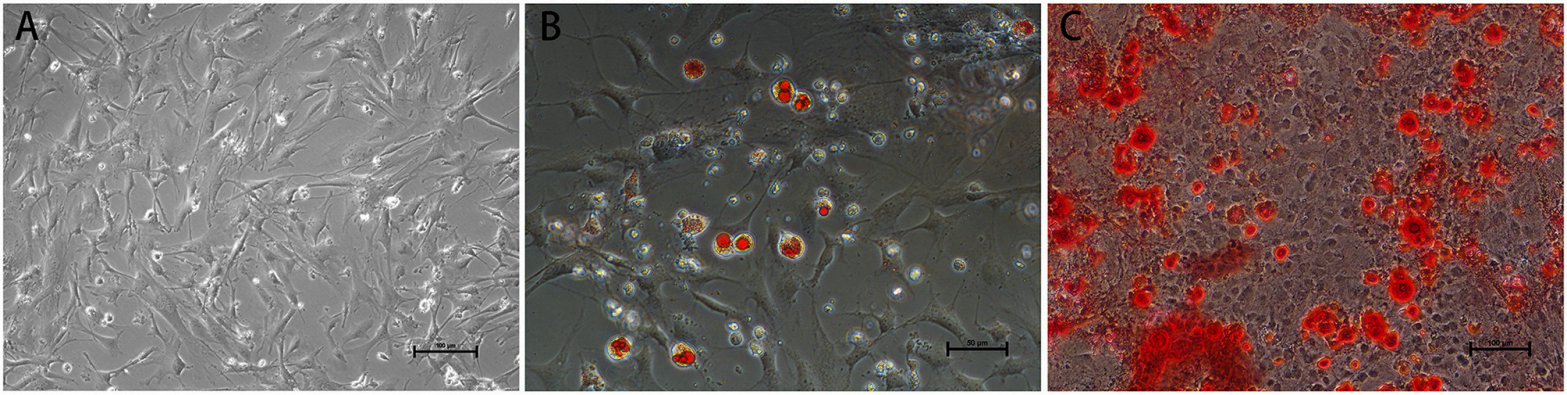
Morphology and differentiation induction of MSCs. **(A)** Typical fibroblast-like cell morphology of MSCs in culture. Scale bar = 100 μm. **(B)** Oil red staining demonstrating fat drops in cell bodies following 2 w adipogenic induction. Scale bar = 50 μm. **(C)** Alizarin red staining demonstrating calcium deposition in cell matrix following 2 w osteogenic induction. Scale bar = 100 μm.

**Figure 2:**
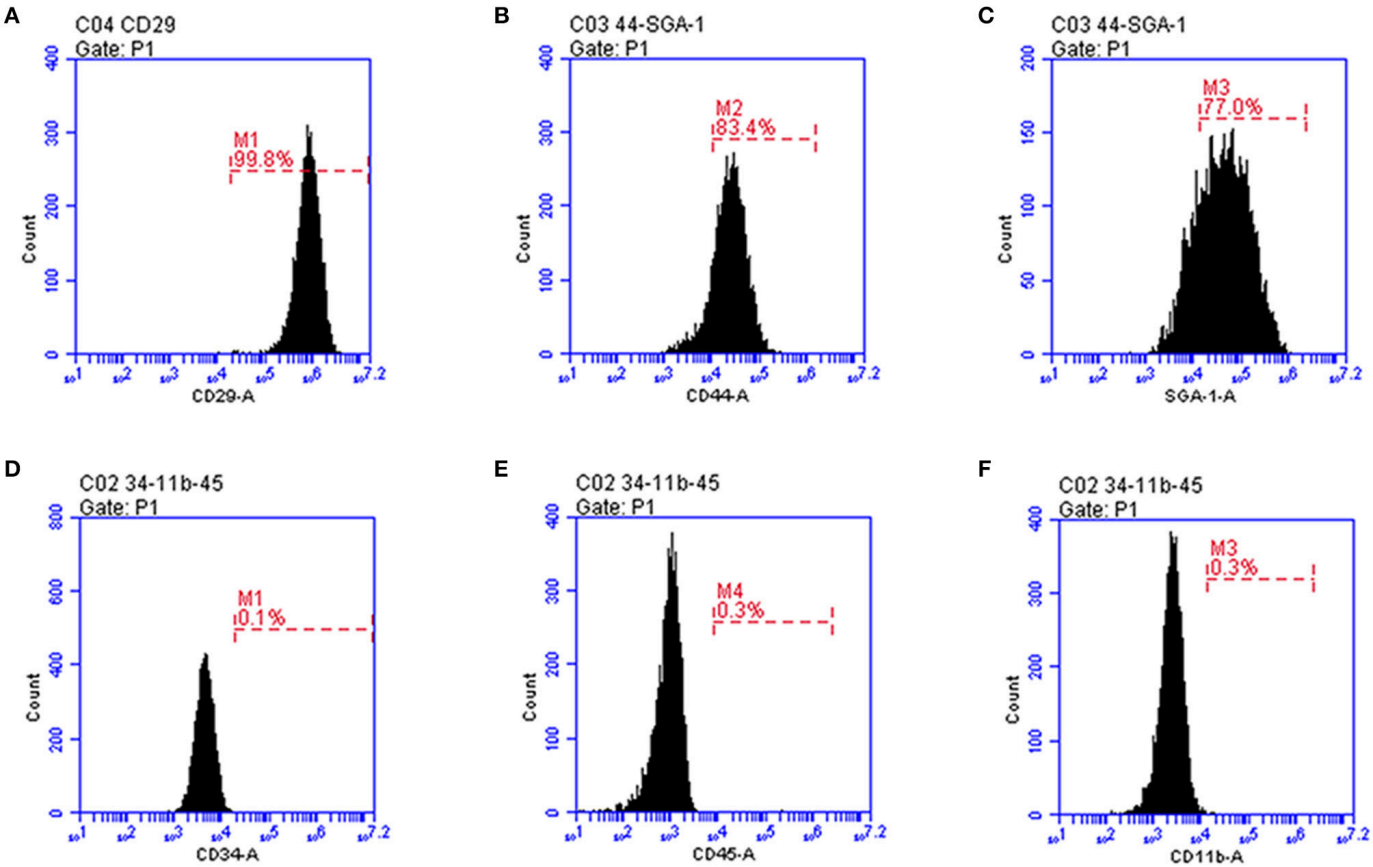
Flow cytometry characterization of MSC expressed markers. **(A)** CD29; **(B)** CD44; **(C)** SGA-1; **(D)** CD34; **(E)** CD45; **(F)** CD11b.

Before transplantation of MSCs for restoring the neurological function after ischemia, we first confirmed that the mouse fetus-derived MSCs had the features of stem cells. The MSCs showed typical fibroblast-like cell morphology in culture (Figure 1). Flow cytometric analysis confirmed that CD29, CD44 and SGA1 surface markers in MSCs were positive, whereas CD34, CD45, and CD11b surface markers were negative (Figure 2). The cell matrix exhibited nodus-like calcium deposition following the alizarin red staining and enlarged and fused fat drops in some cell bodies appeared salmon pink following oil red staining after 2 weeks induction, suggesting that these MSCs had the ability to differentiate into osteoblasts and adipocytes (Figure 1).

### Effect of borneol and MSCs transplantation on neurological deficits

**Figure 3:**
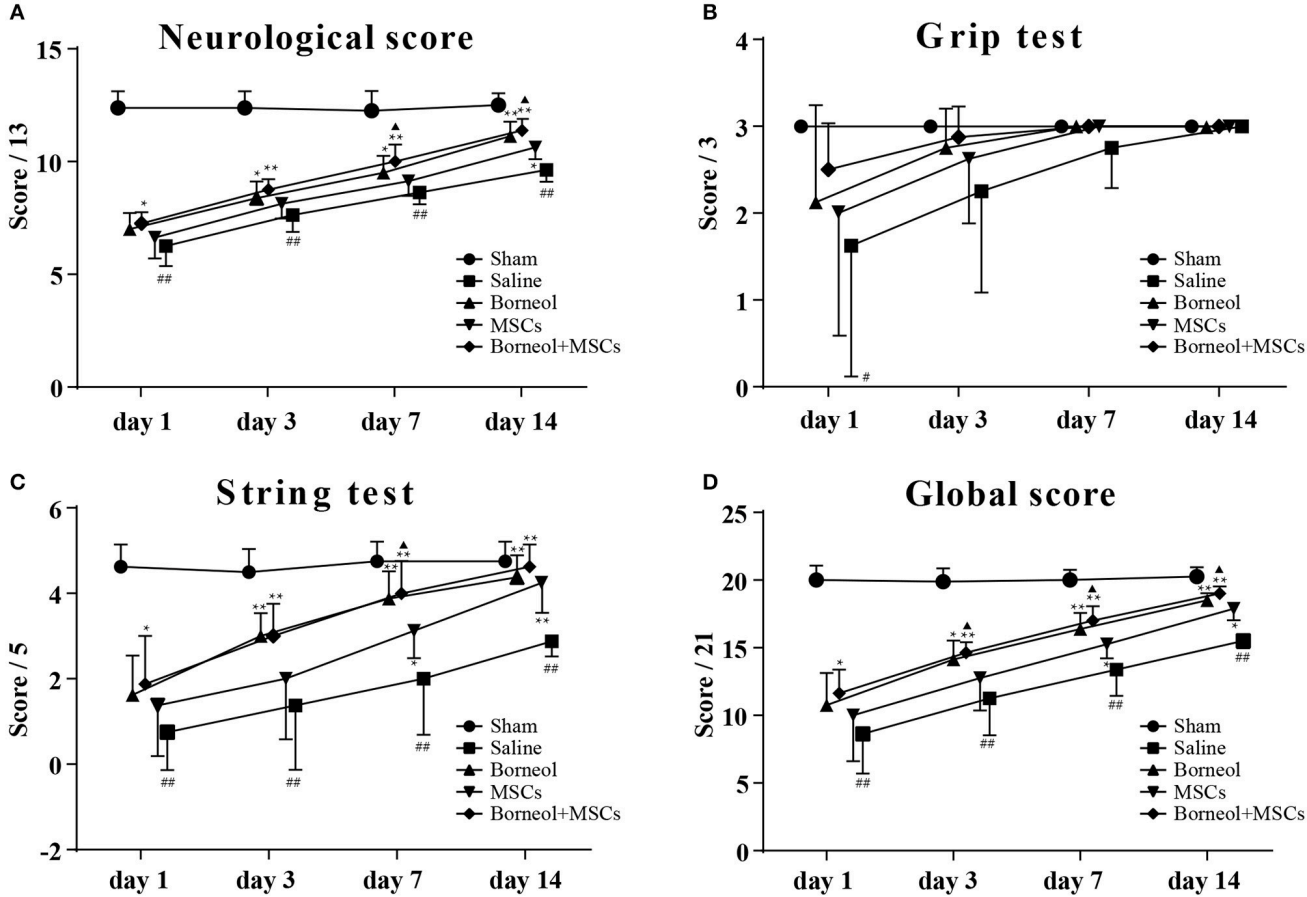
Effect of borneol and MSCs transplantation on neurological deficits. At 1, 3, 7, and 14 d after MSCs transplantation, neulogical deficits were evaluated by **(A)** the neurological score, **(B)** the grip test, **(C)** the string test and **(D)** the global score. Data were expressed as mean ± *SD*. ^##^*P* < 0.01 compared with sham group; ^*^P < 0.05, ^**^P < 0.01 compared with MCAO plus saline group; ^▴^*P* < 0.05, compared with MSCs group.

Compared with sham group, mice in MCAO group showed significant deficits in neurological score (*P* < 0.01; Figure 3A), string test (*P* < 0.01; Figure 3C) and global score (*P* < 0.01; Figure 3D), no matter at 1, 3, 7, or 14 d after ischemic stroke except for grip test (Figure 3B). With administration of borneol, mice in MCAO plus borneol group showed significant improvement in neurological score at 3 d (*P* < 0.01; Figure 3C; *P* < 0.05; Figure 3D), 7 d (*P* < 0.05; Figure 3A) and 14 d (*P* < 0.01; Figure 3A), in string test and global score at 3 d (*P* < 0.01, Figure 3C; *P* < 0.05; Figure 3D), 7 d (*P* < 0.01; Figures 3C,D) and 14 d (*P* < 0.01; Figures 3C,D) compared with MCAO group. Similarly, mice in MCAO plus MSCs group showed significant improvement in neurological score at 14 d (*P* < 0.05; Figure 3A), in string test and global score at 7 d (*P* < 0.05; Figures 3C,D), 14 d (*P* < 0.01; Figure 3C; *P* < 0.05; Figure 3D). Compared with MCAO group, mice in borneol plus MSCs group showed significant improvement in neurological score, string test and global score, no matter at 1 d (*P* < 0.05; Figures 3A,C,D), 3 d (*P* < 0.01, Figures 3A,C,D), 7 d (*P* < 0.01; Figures 3A,C,D) or 14 d (*P* < 0.01; Figures 3A,C,D). Moreover, combination of borneol administration and MSCs transplantation brought more benefit than MSCs transplantation alone on neurological score at 7 d (*P* < 0.05; Figure 3A) and 14 d (*P* < 0.05; Figure 3A), on string test at 7 d (*P* < 0.05; Figure 3C), and on global score at 3 d (*P* < 0.05; Figure 3D), 7 d (*P* < 0.05; Figure 3D) and 14 d (*P* < 0.05; Figure 3D).

### Effect of borneol and MSCs transplantation on infarct volume

**Figure 4:**
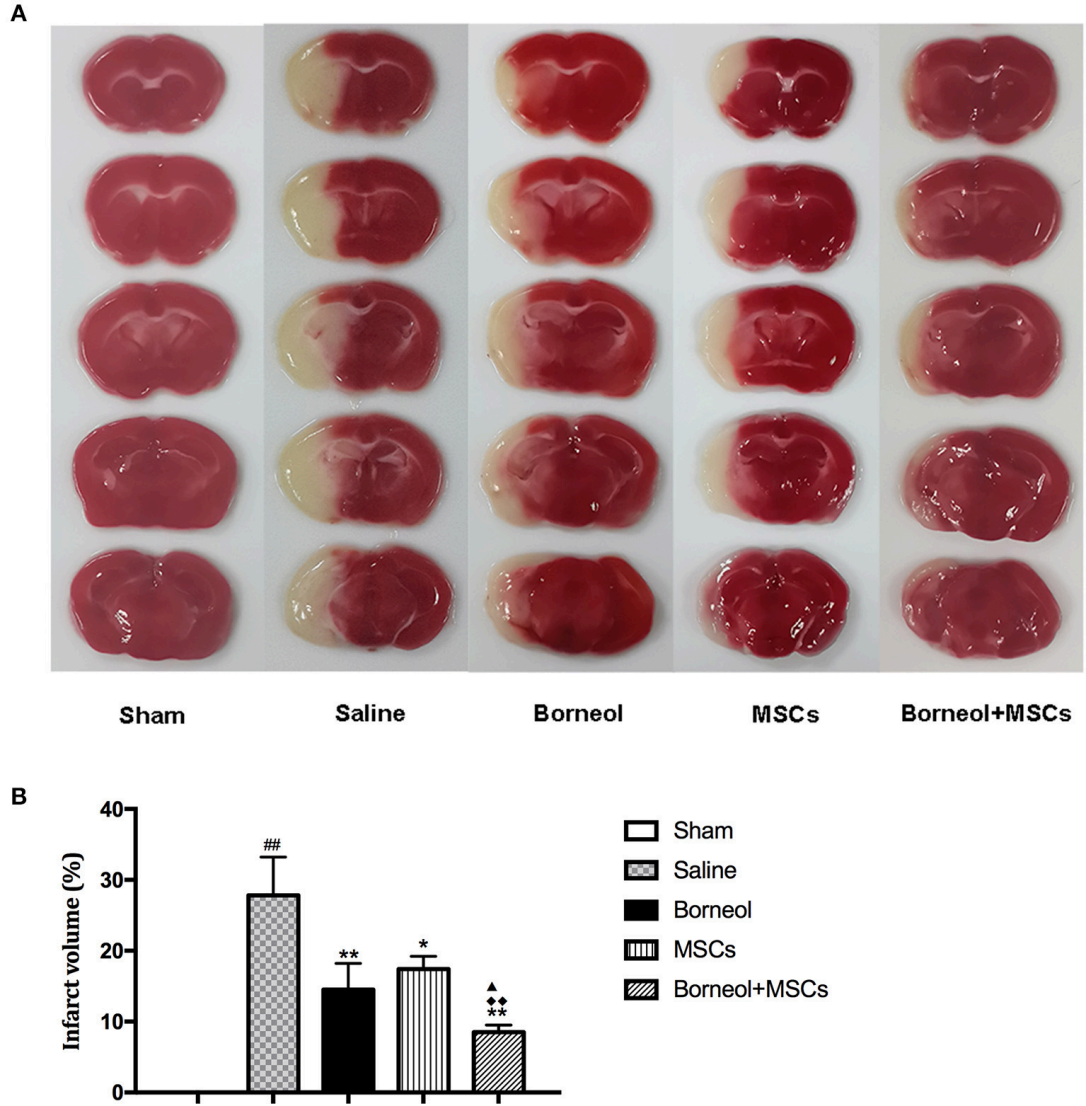
Effect of borneol and MSCs transplantation on infarct volume at 7 d after MSCs transplantation. **(A)** TTC-stained brain slices from sham, MCAO plus saline, borneol, MSCs, and borneol plus MSCs group. **(B)** Quantitative analysis of infarct volume. Data were expressed as mean ± *SD*. ^##^*P* < 0.01 compared with sham group; ^*^P < 0.05, ^**^P < 0.01 compared with MCAO plus saline group; ^▴^*P* < 0.05 compared with borneol group; ^♦♦^*P* < 0.01 compared with MSCs group.

TTC staining was conducted to detect brain tissue damage caused by ischemic reperfusion. Infarction area was showed in sham, MCAO plus saline, borneol, MSCs, and borneol plus MSCs group, respectively (Figure 4A). Compared with sham group, MCAO plus saline mice showed significant increase in cerebral infarct volume (27.8 ± 5.4; *P* < 0.01; Figure 4B). Either borneol administration alone (14.5 ± 3.7; *P* < 0.01), MSCs transplantation alone (17.4 ± 1.8; *P* < 0.05) or combination of these two (8.5 ± 1.0; *P* < 0.01) significantly decreased the infarct volume of ischemic mice, and borneol plus MSCs brought more benefit on infarct volume compared with borneol administration alone and MSCs transplantation alone (*P* < 0.05 or *P* < 0.01; Figure 4B).

### Effect of borneol and MSCs transplantation on cell death

**Figure 5:**
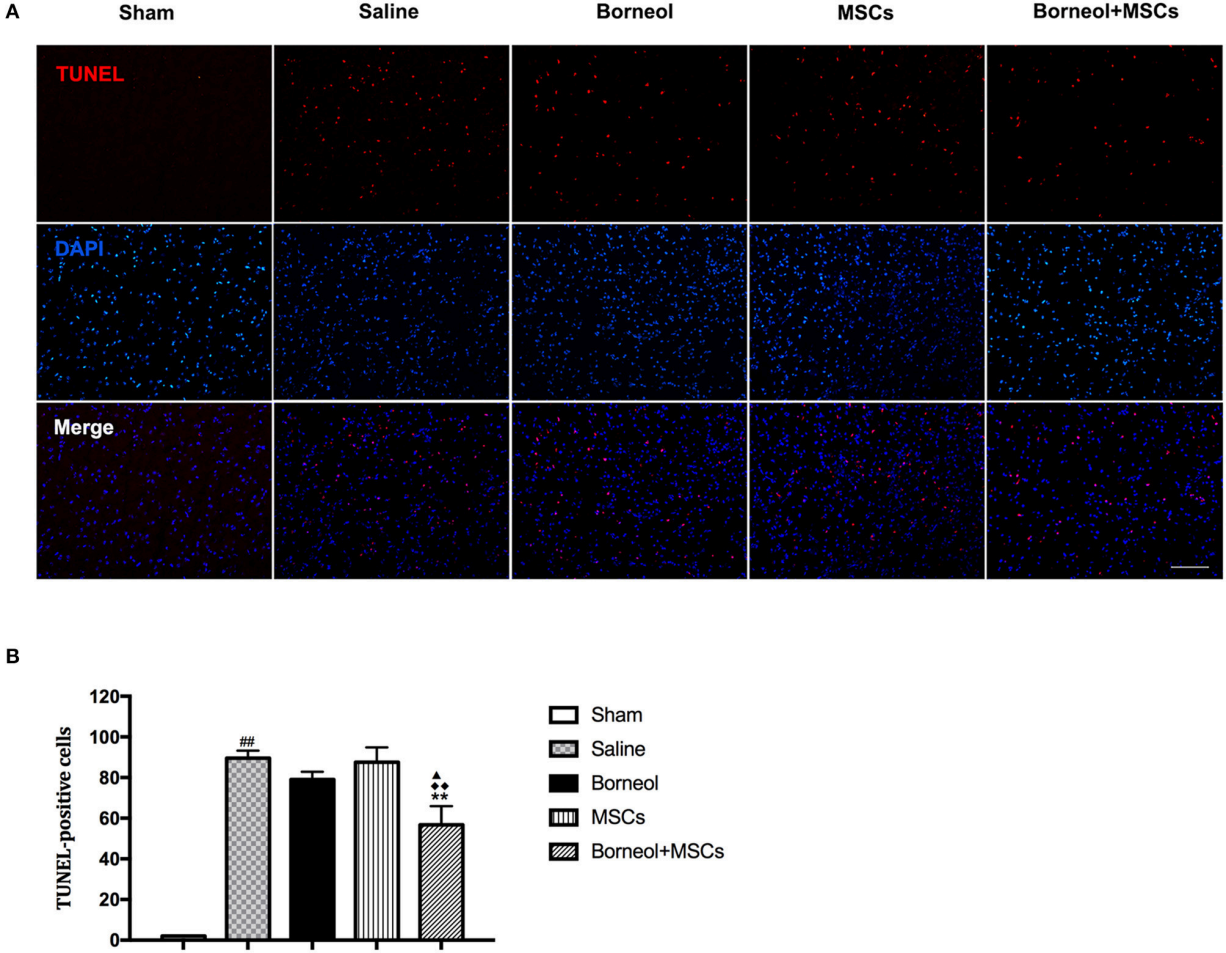
Effect of borneol and MSCs transplantation on cell death at 7 d after MSCs transplantation. **(A)** Photomicrographs (^*^200) of apoptotic cells. **(B)** Quantitative analysis of the number of TUNEL positive cells. Data were expressed as mean ± *SD*. ^##^*P* < 0.01 compared with sham group; ^**^P < 0.01 compared with MCAO plus saline group; ^▴^*P* < 0.05 compared with borneol group; ^♦♦^*P* < 0.01 compared with MSCs group.

Cell death was evaluated by detecting TUNEL positive cells at the infarct zone of stroke mice. Photomicrographs of apoptotic cells were showed in sham, MCAO plus saline, borneol, MSCs, and borneol plus MSCs group, respectively (Figure 5A). As show in Figure 5B, TUNEL positive cells was significantly increased in MCAO plus saline mice (89.6 ± 3.64) comparing with sham group (2.0 ± 0; *P* < 0.01). Borneol and MSCs treatment alone could slightly decreased TUNEL positive cells, with the amount of TUNEL positive cells reaching to 79.0 ± 3.87 and 87.6 ± 7.30, respectively. However, there was no significant statistical difference relative to MCAO group (*P* > 0.05; Figure 5B). The combination of these two (56.8 ± 9.15) could significantly attenuated TUNEL positive cells comparing with MCAO group (*P* < 0.01), borneol group (*P* < 0.05) and MSCs group (*P* < 0.01) (Figure 5B).

### Effect of borneol and MSCs transplantation on neurogenesis in infarct lesion

**Figure 6:**
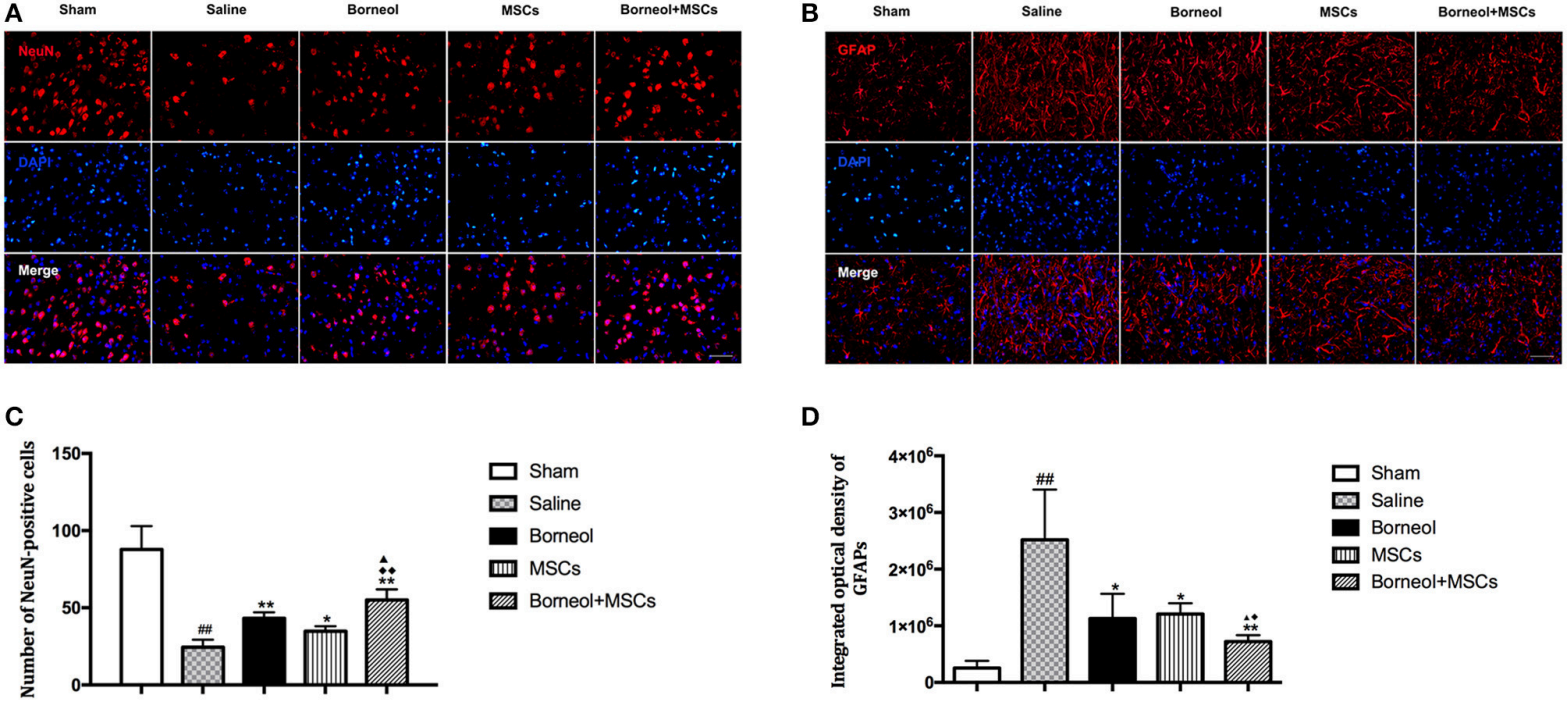
Effect of borneol and MSCs transplantation on neurons and astrocytes at 14 d after MSCs transplantation. **(A)** Photomicrographs (^*^400) of NeuN positive cells in infarct zone. **(B)** Photomicrographs (^*^400) of GFAP positive cells in the lesion border. **(C)** Quantitative analysis of the number of NeuN positive cells. Data were expressed as mean ± *SD*. ^##^*P* < 0.01 compared with sham group; ^*^P < 0.05, ^**^P < 0.01 compared with MCAO plus saline group; ^▴^*P* < 0.05 compared with borneol group; ^♦♦^*P* < 0.01 compared with MSCs group. **(D)** Quantitative analysis of the number of GFAP positive cells. Data were expressed as mean ± *SD*. ^##^*P* < 0.01 compared with sham group; ^*^P < 0.05, ^**^P < 0.01 compared with MCAO plus saline group; ^▴^*P* < 0.05 compared with borneol group; ^♦^*P* < 0.05 compared with MSCs group.

To investigate the effect of borneol and MSCs on neurogenesis *in vivo*, NeuN^+^ cells (Figure 6A) and GFAP^+^ cells (Figure 6B) in the infarct cortex were assessed by immunofluorescence in sham, MCAO plus saline, borneol, MSCs, and borneol plus MSCs group, respectively. Immunofluorescence study revealed that NeuN^+^ cells decreased significantly in ischemic mice (24.4 ± 4.9, *P* < 0.01; Figure 6C), while borneol administration alone (43.2 ± 3.9, *P* < 0.01; Figure 6C), MSCs transplantation alone (34.8 ± 3.3, *P* < 0.05; Figure 6C) and combination of these two (55.0 ± 6.9, *P* < 0.01; Figure 6C) all significantly increased the number of NeuN^+^ cells compared with MCAO group. Moreover, borneol plus MSCs had a better effect on NeuN^+^ cells improvement compared with either borneol administration alone (*P* < 0.05; Figure 6C) or MSCs transplantation alone (*P* < 0.01; Figure 6C).

The IOD of GFAP increased markedly in MCAO group, whereas after borneol administration (1,129,171 ± 436,009, *P* < 0.05; Figure 6D), MSCs transplantation (1,207,876 ± 191,142, *P* < 0.05; Figure 6D) and borneol plus MSCs (722,058 ± 111,587, *P* < 0.01; Figure 6D), IOD of GFAP decreased significantly compared with MCAO group (2,518,680 ± 885,565). Compared with either borneol administration alone (*P* < 0.05; Figure 6D) or MSCs transplantation alone (*P* < 0.05; Figure 6D), borneol plus MSCs group slightly reduce the number of GFAP^+^ cells.

## Discussion

The present study showed that intravenous infusion of MSCs in combination with borneol by gavage could more effectively improve the neurological deficits, reduce infarction volume and cell apoptosis, and enhance neurogenesis than that of MSCs alone. These data supported the hypothesis that combination use of borneol and stem cells might provide an additive neuroprotective effect on cerebral ischemia.

Recent studies have demonstrated the efficacy of stem cell-based therapy in experimental ischemic stroke (Rodríguez-Frutos et al., 2016). Although stem cell therapy was considered to be a promising regenerative strategy for stroke patients, its efficacy was still controversial within current clinical trials (Bang, 2016). An open-label, observer-blinded, randomized clinical trial with intravenous infusion of 5 × 10^7^ autologous MSCs in patients with subacute ischemic stroke (seventh admission day with stable course) showed long-term beneficial effects in 5-year follow-up (Lee et al., 2010). Conversely, in a phase II, multicenter, parallel group, randomized trial, intravenous administration of a mean of 2.8 × 10^7^ autologous bone marrow mononuclear cells in subacute phase (median of 18.5 days after stroke onset) of stroke patients failed to show its effectiveness (Prasad et al., 2014). The inconsistent results might be attributed to the state of patients, cell dosage, therapeutic window, etc. Further studies concerning the effect of stem cell-based therapy on cerebral ischemia were still needed.

Recently, a hypothesis has been proposed that combinations of neuroprotective agents may have additive or multiplicative beneficial effects (Aronowski et al., 2003; Liu et al., 2009). For instance, either administration of ethanol or caffeine alone showed little effect, while the combination of those two was effective for treatment of ischemic stroke (Aronowski et al., 2003). Borneol and MSCs were both regarded as neuroprotective agents, for the reason that borneol could increase drug penetration into brain while MSCs could provide trophic support. However, whether they have the additive effect on cerebral ischemic protection was unknown. Thus in the present study, we evaluated whether borneol could enhance the neuroprotective effect of MSCs in the mouse MCAO model.

Borneol in combination with MSCs or MSCs alone reduced lesion volume and cell apoptosis as assayed by histology, but this protective effect was greater in the borneol plus MSCs group. Cerebral ischemia/reperfusion is a dynamic process, which can cause a non-recoverable loss of cell viability, further leading to neuronal death (Hossmann, 2006). TUNEL marking of cell death was significantly decreased in the infarct zone of the MSCs group. This finding was consistent with previous studies that reported reduction in apoptotic cell number after 2 × 10^6^ BM-MSCs administration at 90 mins after ischemia (Gutiérrez-Fernández et al., 2011) and 2 × 10^6^ AD-MSCs administration at 0, 12, 24 h after ischemia (Leu et al., 2010). When combining with borneol, apoptotic cells reduced more significantly compared with MSCs group, suggesting that borneol might strengthen the protective effect of MSCs on cell viability and the reduction of infarct volume might be attributed to the reduction in the number of apoptotic cells to some extent.

Several factors are probably involved in achieving the benefits of MSCs in the ischemic brain, among which the improvement of functional recovery and infarct volume reduction could believe to be the consequence of the activation of endogenous repair mechanisms such as proliferation and migration of endogenous neural stem cells (Zhang and Chopp, 2009; Gutiérrez-Fernández et al., 2011). The effect of endogenous neurogenesis would further be enlarged by the trophic support provided by MSCs in the ischemic brain. As found in this study, MSCs used alone could significantly increase the number of NeuN^+^ cells in infarct area. Moreover, combination of borneol and MSCs had a better effect on NeuN^+^ cells improvement compared with MSCs used alone. However, the IOD of GFAP^+^ cells were significantly lower in the borneol plus MSCs group. Astrocytes were found mainly in the corpus callosum of the ischemic hemisphere and sporadically at the lesion border at the beginning of ischemic reperfusion. With time the reactive astrocytes infiltrated into the lesion core and formed a visible dense “wall” at the lesion border which was indicated for a mature glial scar as a physical barrier that prevented the spread of the inflammation (Hamzei Taj et al., 2016). Recent study reported that this astrocytic scar could also enable spontaneous axon regrowth through severe lesions (Rolls et al., 2009; Anderson et al., 2016). Interestingly, inconsistent with the effect of borneol plus MSCs on NeuN^+^ cells improvement, we found that GFAP^+^ density slightly decreased by the joint application of borneol and MSCs. This change might enable more trophic factors to infiltrate into the lesion core and thus contributed to neuron regeneration.

In summary, the present study demonstrated that borneol and MSCs had the additive neuroprotective effect on cerebral ischemia. However, the supposed effect of borneol on the improved MSC penetration still needs further direct evidence.

## Author contributions

X-GZ, CS, X-YB, QT, J-ZZ, X-FW, X-CT, TX, JL, G-QZ, and YW: designed the study; X-GZ and CS: performed the experiments; X-GZ, CS, X-YB, QT, J-ZZ, X-FW, X-CT, and TX: analyzed the data; X-GZ, X-FW, and X-CT: prepared the figures; X-GZ: drafted the manuscript; G-QZ, JL, and YW: edited the manuscript; All authors contributed to writing of the final version of this manuscript.

### Conflict of interest statement

The authors declare that the research was conducted in the absence of any commercial or financial relationships that could be construed as a potential conflict of interest.
